# Nanostructured Chitosan/Maghemite Composites Thin Film for Potential Optical Detection of Mercury Ion by Surface Plasmon Resonance Investigation

**DOI:** 10.3390/polym12071497

**Published:** 2020-07-04

**Authors:** Nurul Illya Muhamad Fauzi, Yap Wing Fen, Nur Alia Sheh Omar, Silvan Saleviter, Wan Mohd Ebtisyam Mustaqim Mohd Daniyal, Hazwani Suhaila Hashim, Mohd Nasrullah

**Affiliations:** 1 Department of Physics, Faculty of Science, Universiti Putra Malaysia, UPM Serdang 43400, Selangor, Malaysia; illyafauzi97@gmail.com (N.I.M.F.); hazwanisuhaila@gmail.com (H.S.H.); 2 Functional Devices Laboratory, Institute of Advanced Technology, Universiti Putra Malaysia, UPM Serdang 43400, Selangor, Malaysia; nuralia.upm@gmail.com (N.A.S.O.); silvansaleviter94@gmail.com (S.S.); wanmdsyam@gmail.com (W.M.E.M.M.D.); 3 Faculty of Civil Engineering Technology, Universiti Malaysia Pahang (UMP), Gambang 26300, Kuantan, Pahang, Malaysia; nasrul.ump@gmail.com

**Keywords:** chitosan, maghemite, optical, mercury ion, surface plasmon resonance

## Abstract

In this study, synthesis and characterization of chitosan/maghemite (Cs/Fe_2_O_3_) composites thin film has been described. Its properties were characterized using Fourier transform infrared spectroscopy (FTIR), atomic force microscopy (AFM) and ultraviolet-visible spectroscopy (UV-Vis). FTIR confirmed the existence of Fe–O bond, C–N bond, C–C bond, C–O bond, O=C=O bond and O–H bond in Cs/Fe_2_O_3_ thin film. The surface morphology of the thin film indicated the relatively smooth and homogenous thin film, and also confirmed the interaction of Fe_2_O_3_ with the chitosan. Next, the UV-Vis result showed high absorbance value with an optical band gap of 4.013 eV. The incorporation of this Cs/Fe_2_O_3_ thin film with an optical-based method, i.e., surface plasmon resonance spectroscopy showed positive response where mercury ion (Hg^2+^) can be detected down to 0.01 ppm (49.9 nM). These results validate the potential of Cs/Fe_2_O_3_ thin film for optical sensing applications in Hg^2+^ detection.

## 1. Introduction

Organic polymeric materials made up of many repeating monomer units have made a significant impact on biological and biomedical research activities because of the flexibility and the ease of fabrication [1]. One of the well-known organic polymeric materials is chitosan, easily derived from partial deacetylation of chitin with a degree of 50% or greater [2,3,4]. To be more specific, chitosan is a family of linear polysaccharide as a part of glucosamine and N-acetyl glucosamine units linked via β-1,4 glucosidic bonds [5,6]. Chitosan contains three types of reactive functional groups, primary amine groups and primary and secondary hydroxyl groups, respectively, at positions C-2, C-3 and C-6. Among the three types of functional groups, the primary amine groups at C-2 positions are the most favorable sites interacting with the biological molecules, metal ions and organic halogen substances. Taking the advantages of chitosan with high absorption capacity and high biocompatibility, chitosan is known as an ideal substrate for enzyme immobilization [7]. Other excellent advantages of chitosan including non-toxicity, great film-forming ability, powerful adhesion property and high mechanical strength, offers great room for sensor applications [8,9,10]. However, the problem of poor stableness of chitosan because of the hydrophilic character and pH sensitivity restricts its application [11,12]. Previous reports showed that the stability of chitosan could be improved by combining with oxide or metal oxides and the product can be effectively used as recognition elements for chemical sensors and biosensors [13,14,15].

Iron (III) oxide or ferric oxide is the inorganic compound with the Fe_2_O_3_ formula, which varies in color depending on its phase [16]. Fe_2_O_3_ materials have four polymorphs phases such as α-Fe_2_O_3_ (hematite), β-Fe_2_O_3_, γ-Fe_2_O_3_ (maghemite) and ε-Fe_2_O_3_ [17,18]. The differences of the phases are known from their originality, for examples, hematite and maghemite are naturally obtained and the other two of phases are synthesized in laboratory [19,20]. Among the phases, γ-Fe_2_O_3_ is one of the chief interests. It is the second most common sustainable form of Fe_2_O_3_, known as completely oxidized magnetite. Maghemite has a high curie temperature, but has a lower saturation magnetization at room temperature and a supermagnetism property that makes it quite efficient in removing heavy metal pollutants from water [21,22]. Moreover, it is believed that Fe_2_O_3_ can improve and provide better mechanical properties to chitosan [23].

Accumulation of heavy metals in water and food production, primarily mercury (Hg) is the most hazardous heavy-metal pollutants even at a very low concentration. The most toxic chemical forms of Hg are ionic Hg (Hg^2+^), causes serious damage to human health such as brain damage, immune dysfunction and paralysis [24,25,26]. Therefore, the removal and detection of Hg^2+^ in the aqueous environment are of great significance [27,28,29,30,31]. Among the existing optical techniques to detect Hg^2+^ are colorimetric, fluorescent, chemosensor, electrochemiluminescence (ECL) and photoluminescent (PL) [32,33,34]. Though these techniques are widely used, they encounter from many drawbacks, such as high instrument operating costs, repetitive pretreatment procedures and long initiation times [35].

Corresponding to the previous methods, surface plasmon resonance (SPR) proposed a cost-effective, label-free detection method for convenient usage, rapid detection and excellent sensitivity and selectivity to heavy metal ions [36,37,38,39,40]. Since enormous efforts devoted to creating sensors with high sensitivity to Hg^2+^ are greatly needed currently, selection of the metallic layer such as the gold layer is an important aid in producing higher sensor sensitivity in SPR [41]. Over the last decade, the surface SPR technique has emerged as an effective optical technique for various applications including detection of heavy metal ions [42,43,44,45,46,47,48,49,50,51]. Unfortunately, the main problem to detect optically the heavy metal ions solution is the similar refractive indices of heavy metal ions for lowest concentration, which eventually becomes the goal of researchers. Hence, many researchers have dedicated their time to develop chitosan-based materials onto SPR interfaces in lowering the detection limit of Hg^2+^, specifically [52,53,54]. A recent study documented the utilization of polypyrrole-chitosan/nickel-ferrite nanoparticles as an active layer to a prism-based on SPR technique for Hg^2+^ sensing, which reached a limit of detection (LOD) as low as 1.94 µM [54]. Other recent studies using chitosan-based materials as sensing layers for the detection of Hg^2+^ by SPR are summarized in Table 1. It is of interest to further improve the LOD using chitosan-based SPR sensor.

To the best of our knowledge, the study for Cs/γ-Fe_2_O_3_ composite to detect Hg^2+^ using the SPR technique is not reported yet. There is also a lack of studies on the structural and optical properties of these composites. Therefore, an effort was made to apply the chitosan/γ-Fe_2_O_3_ thin film onto a thin gold surface, as a novel active layer for the SPR technique in sensing Hg^2+^ as low as nanomolar. Besides, the studies of structural and optical properties of Cs/γ-Fe_2_O_3_ thin film on the gold surface are also reported and explored.

## 2. Materials and Methods 

### 2.1. Reagent and Materials

The Fe_2_O_3_ was purchased from R&M Marketing, Essex, U.K. The medium molecular weight chitosan and acetic acid were purchased from Aldrich (Saint louis, MO, USA). Standard solution of Hg^2+^ with concentration of 1000 ppm was purchased from Merck (Darmstatd, Germany).

### 2.2. Preparation of Chemical

Firstly, 50 mL distilled water was added into Fe_2_O_3_ (4 mg/mL). Then 10 mL of NH_3_ (25%) and 0.615 mg of ethylenediaminetetra acetic acid (EDTA) was added as precipitation agent and as capping agent to the solution with stirring respectively. The reaction was allowed to proceed for 1 h at 50 °C with constant stirring. Finally, the black precipitate of nano-Fe_2_O_3_-EDTA formed and it was rinsed with distilled water and left to dry 80 °C for 3 h. For chitosan preparation, 1% acetic acid was prepped by diluting stock 1 mL acetic acid with deionized water in 100 mL volumetric flask. Next, 400 mg medium molecular weight chitosan that was acquired from Aldrich was dissolved in 50 mL of 1% aqueous acetic acid and the solution vigorously stirring to ensure powder chitosan dissolved completely. To produce the nanostructured chitosan/maghemite (Cs/Fe_2_O_3_) composites, 30 mg Fe_2_O_3_ capped EDTA was dispersed in 10 mL of 0.1% in chitosan solution and sonicated in room temperature for 15 min. The Hg^2+^ standard solution with a concentration of 1000 ppm was diluted with deionized water to produce Hg^2+^ solutions with concentrations of 0.01, 0.05, 0.08, 0.1 and 0.5 ppm [55,56].

### 2.3. Preparation of Thin Film

To begin, glass slips (24 mm × 24 mm × 0.1 mm, Menzel-Glaser, Braunschweig, Germany), as a substrate, were coated with a thin layer of gold with thickness 50 nm using SC7640 sputter coater [57]. Next, approximately 0.55 mL of the chitosan, Fe_2_O_3_ and Cs/Fe_2_O_3_ composites solution was set separately on the surface of the gold coated glass slip. Then the glass slips were spun at 6000 rev min for 30 s using the Specialty Coating System, P-6708D (Inc. Medical Devices, Indianapolis, IN, USA) to produce the chitosan, Fe_2_O_3_ and Cs/Fe_2_O_3_ composites thin films.

### 2.4. Instrumental

**Figure 1:**
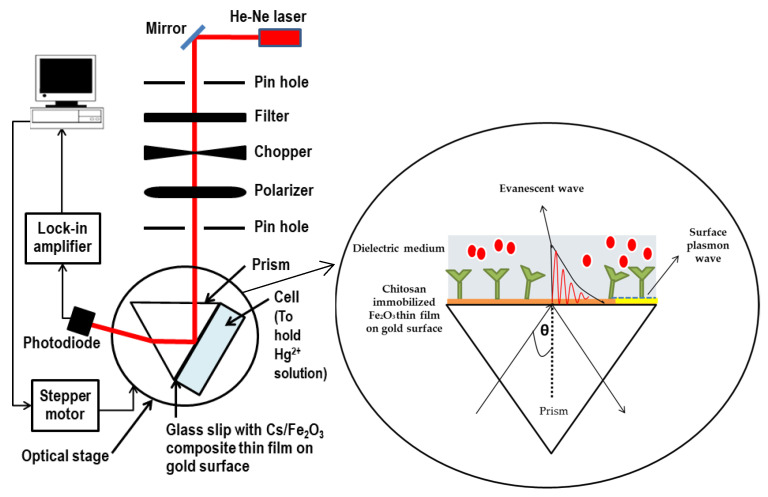
Optical setup of surface plasmon resonance spectroscopy.

Fourier transform infrared (FTIR) spectra for each surface modification of thin films were recorded in the transmittance mode using a Perkin-Elmer spectrophotometer (Waltham, MA, USA) under the wavelength range 400–4000 cm^−1^. The absorbance spectra of the films were recorded from 200 to 500 nm using UV-Vis-NIR spectroscopy (UV-3600 Shimadzu, Kyoto, Japan). The optical band gap energy was calculated using the data obtained. Atomic force microscopy (AFM) analysis was carried out using Qscope 250, Qesant Instrument Corporation (Quesant, CA, USA) in intermittent mode to study the topography and height of Cs/Fe^2^O^3^ thin film. An optical-based sensing method based on surface plasmon resonance (SPR) was designed to identify the potential of the Cs/Fe_2_O_3_ thin film to detect Hg^2+^. Figure 1 shows the schematic diagram of the SPR instrument setup [58,59,60,61]. The SPR experiment was carried out by inserting Hg^2+^ solutions with different concentration varied from 0.01 to 0.5 ppm. It was injected one after another into the cell to bind with Cs/Fe_2_O_3_ thin film coated onto gold surface thin film. The SPR curve and resonance angle for all concentrations was monitored and recorded.

## 3. Results and Discussion

### 3.1. FTIR Analysis

**Figure 2:**
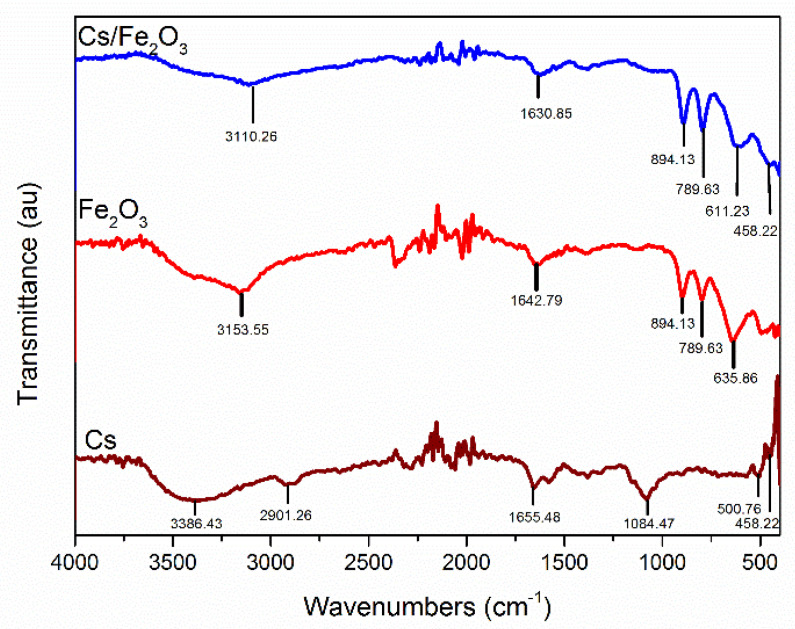
FTIR spectrum for chitosan, Fe_2_O_3_ and Cs/Fe_2_O_3_ thin films.

FTIR spectroscopy was used to identify the functional groups existed in Cs/Fe_2_O_3_ thin film. The spectrum of chitosan, Fe_2_O_3_ and Cs/Fe_2_O_3_ thin films in the range of 450–4000 cm^−1^ are represented in Figure 2. From the FTIR spectrum of chitosan thin film, the broad absorption band at 3386.43 cm^−1^ can be appointed to the stretching vibration of O–H. A weaker band found at 2901.26 cm^−1^ can be attributed to C–H stretching in chitosan. Another absorption band at 1655.48 cm^−1^ was associated with the presence of the C=O stretching bond. There is an absorption peak at 1084.47 cm^−1^ that corresponds to the C–O group, which indicates the presence of the –COOH group in chitosan thin film. Two more bands at 500.76 cm^−1^ and 458.22 cm^−1^ were assigned to the C–C bond and C–N bond respectively. This finding is well aligned to the previous study by Anas et al. [62].

Next, a particular major peak in the Fe_2_O_3_ thin film was identified with the degree of cation vacancy, ordering between octahedral Fe cation and O atoms [63]. The absorption peak at 789.63 cm^−1^ is a characteristic of maghemite Fe–O stretching vibrations particles. This peak is solely attributed to the high degree of cationic vacancy ordering [64]. The broad band characteristic for bending vibration of water adsorbed on the maghemite’s surface is at 2078.99 cm^−1^. The intense bands at 1642.79 cm^−1^ and 3153.55 cm^−1^ were then assigned to CO_2_ vibration and O–H vibrations, respectively, ratifying the presence of surface γ-Fe_2_O_3_ hydroxyl groups.

In the spectrum of Cs/Fe_2_O_3_, the chitosan does not provide clear absorption bands at a lower wavenumber. This is due to the low percentage of chitosan compared to maghemite in the synthesization process. However, the presence of chitosan can be observed based on the intensity peak. The peak intensity of Cs/Fe_2_O_3_ clearly increased after the sorption of chitosan and Fe_2_O_3_, i.e., at C–H stretching (458.22 cm^−1^), C–C bond (611.23 cm^−1^) and O=C=O stretching (1630.85 cm^−1^). An increase in the peak intensity usually indicates an increase in the sum of the functional group (per unit volume) associated with the molecular bond [65]. On the other hand, a strong absorption band was observed at 789.63 cm^−1^, confirmed the presence of Fe_-_O as the main phase of the Fe_2_O_3_ and a band at 3110.26 cm^−1^ that appointed to the O-H vibration of surface maghemite hydroxyl groups. Overall, the FTIR results showed the increasing peak intensity of Cs/Fe_2_O_3_, which confirmed the physical interaction of chitosan and γ-Fe_2_O_3_ in those composites. 

### 3.2. Surface Morphology

**Figure 3:**
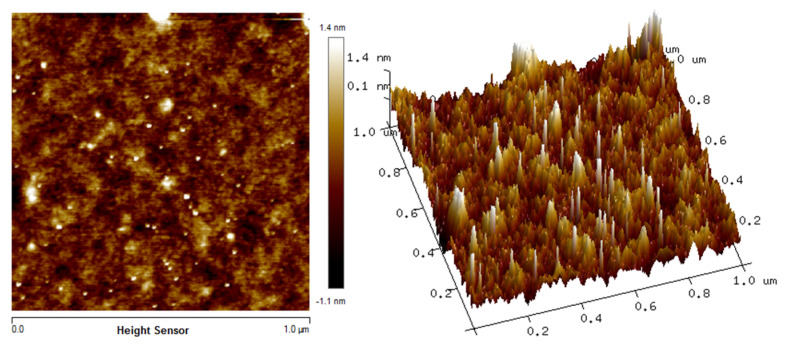
Atomic force microscopy (AFM) image of chitosan thin film.

**Figure 4:**
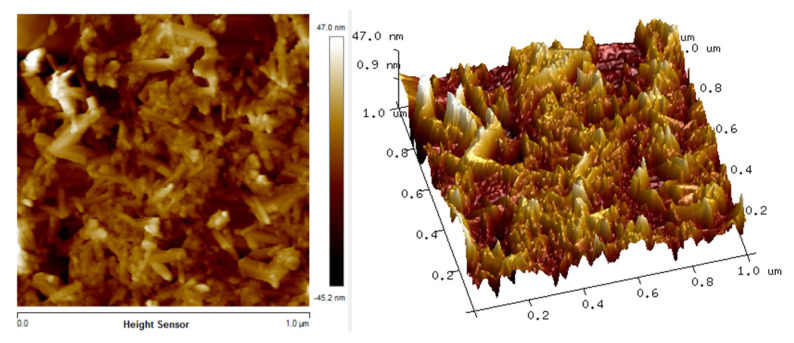
AFM image of Fe_2_O_3_ thin film.

**Figure 5:**
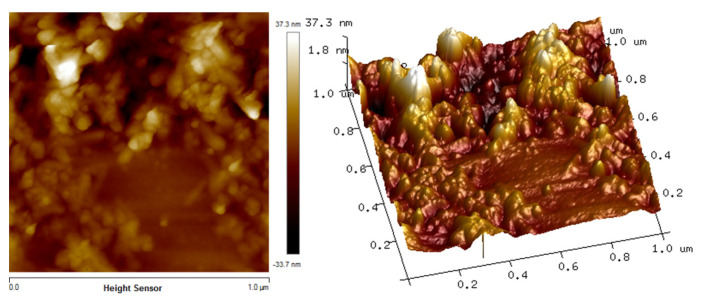
AFM image of Cs/Fe_2_O_3_ thin film.

The in situ atomic force microscopy (AFM) measurements enable the chitosan, Fe_2_O_3_ and Cs/Fe_2_O_3_ adsorption on thin films to be visualized in real time. The AFM images illustrate the topographical in the thin films as shown in Figure 3, Figure 4 and Figure 5. The topographical can be observed by various parameters that exist to quantify the root mean square (rms) roughness of a surface. The RMS roughness value can be calculated from the cross-sectional profile or a surface area [66]. The RMS roughness obtained by chitosan, Fe_2_O_3_ and Cs/Fe_2_O_3_ thin film were 1.4 nm, 47 nm and 37.3 nm, respectively. The magnitude decreased in RMS roughness of Cs/Fe_2_O_3_ thin film compared to Fe_2_O_3_ thin film attributable to the association of two materials, which are chitosan and Fe_2_O_3_. The roughness implies that a smoothening mechanism by surface diffusion [67]. This result indicates that the presence of chitosan can enhance the surface of the thin film. The roughness introduced in the nanostructured maghemite in chitosan thin film intended appropriate form to enhance the thin film as sensing element [68]. This result is in line with the FTIR data, proving the presence of maghemite and chitosan in the Cs/Fe_2_O_3_ thin film based on the RMS roughness.

### 3.3. Optical Studies

**Figure 6:**
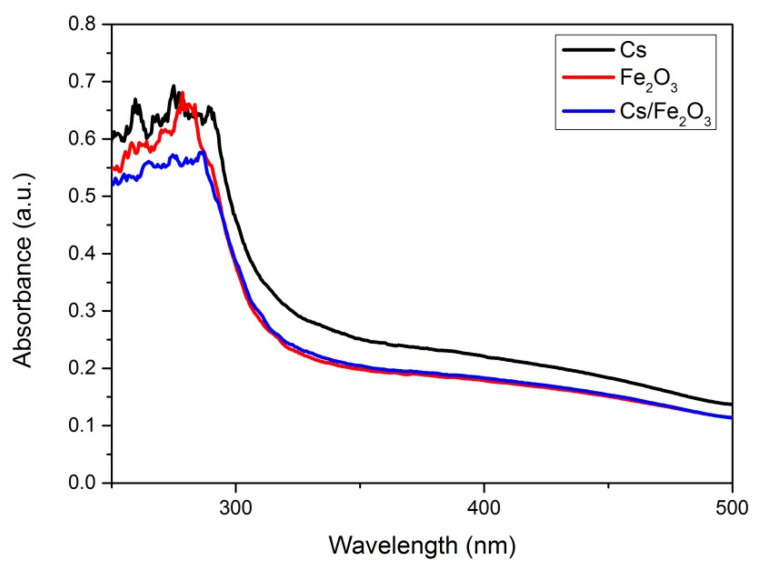
Absorbance spectrum chitosan, Fe_2_O_3_ and Cs/Fe_2_O_3_ thin films.

For the optical properties, the absorbance spectrum of the thin films was observed and measured at wavelength from 250 to 500 nm. The UV-Vis results of chitosan, Fe_2_O_3_ and Cs/Fe_2_O_3_ thin films are shown in Figure 6 it can be spotted that all of the thin film has diverse value of absorbance. From the graph, the absorbance spectra of Cs and Fe_2_O_3_ thin films were slightly higher as compared to the Cs/Fe_2_O_3_ thin film. The maximum absorption wavelength can be observed at 260–300 nm. The absorption peak about 300 nm corresponds to π→π* transitions of C=O [69,70].

The UV-Vis absorbance spectrum was then quantitative analyzed based on the Beer–Lambert law theory. This law refers to a relation between the attenuation of light by a material and its properties, which the monochromatic light (single wavelength) is travelling. Since the amount of the emitted radiation intensity is only dependent on the thickness, *t* and concentration of the solution, the absorbance, *A* of the samples can be collected at a single wavelength, as follows [62]:

(1) $$A=\log_{10}\frac{I_{o}}{I_{t}}$$

The transmittance, *T* of sample is given by the ratio of intensities of the presence *I_t_* and the absence *I_o_* of the sample:

(2) $$T=\frac{I_{t}}{I_{o}}$$

Thus, the absorbance and transmittance can be related by:

(3) $$A=-\log_{10}T$$

Apart from the absorbance, absorbance coefficient is a useful parameter to compare samples with varying thickness. The sample thickness was obtained by using atomic force microscopy. The absorbance coefficient, *α* (in unit of m^−1^) is given by:

(4) $$\alpha =2.303\frac{A}{t}$$

where *t* is the thickness of sample in unit of m. The absorbance coefficient and optical band gap can be related by:

(5) $$\alpha =\frac{k{\left(hv-E_{g}\right)}^{n}}{hv}$$

Rearranging Equation (5) gives:

(6) $${\left(\alpha hv\right)}^{1/n}=k(hv-E_{g})$$

where *hv* is the photon energy, *h* is Plank’s constant, *E_g_* is the optical band gap, *k* is constant and *n* is the transition states, i.e., direct or indirect transitions. Direct transition is transition in which a photon excites an electron from the valence band to the conduction band directly if the momentum of electrons and holes is the same in both bands (conduction and valence). On the other hand, indirect transition is a photon cannot be emitted because the electron must pass through an intermediate state and transfer momentum to the crystal lattice. From these, it can be concluded that the absorption in the thin films corresponds to a direct energy gap. For direct transition, *n* = 1/2 and this value is substituted in Equation (6) and becomes:

(7) $${\left(\alpha hv\right)}^{1/n}=k(hv-E_{g})$$

**Figure 7:**
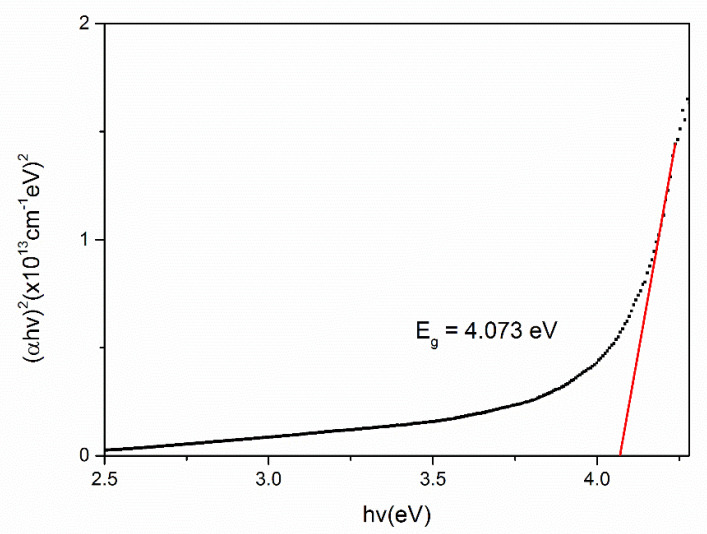
Optical band gap for chitosan thin film.

**Figure 8:**
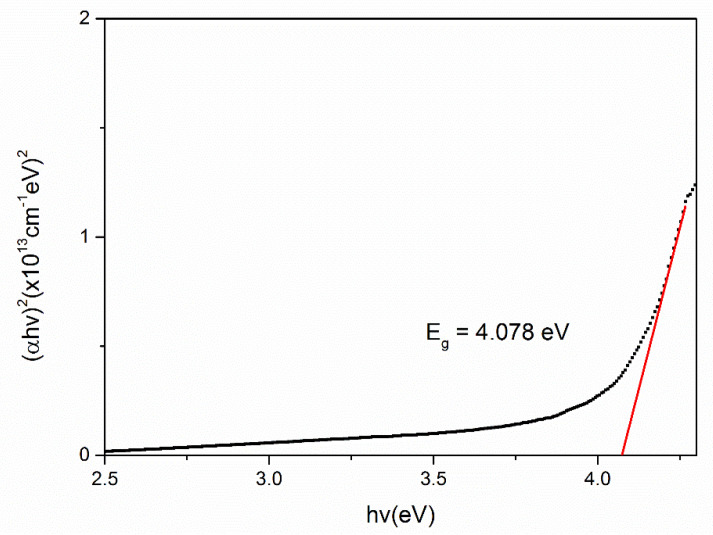
Optical band gap for Fe_2_O_3_ thin film.

**Figure 9:**
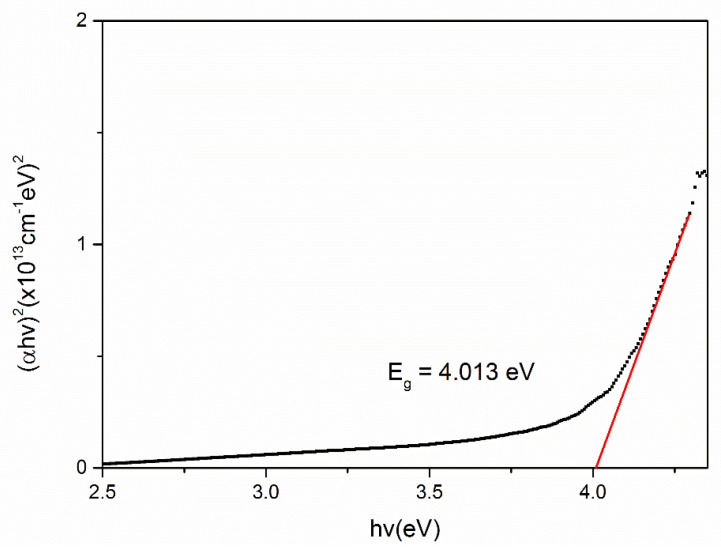
Optical band gap for Cs/Fe_2_O_3_ thin film.

To evaluate the optical band gap, *E_g_* of the chitosan, Fe_2_O_3_ and Cs/Fe_2_O_3_ thin films, the graphs of (*αhv*)^2^ against hv are plotted as shown in Figure 7, Figure 8 and Figure 9, respectively. As a result, the intersection of straight line on the edge was obtained, indicating the direct transition of the optical band gap [71]. The calculated values of the optical band gap were 4.073 eV, 4.078 eV and 4.013 eV for chitosan, Fe_2_O_3_ and Cs/Fe_2_O_3_ thin films respectively (with the corresponding error of ±0.001 eV) [72,73]. This result indicated the maghemite had a band gap energy of 4.078 eV, which was higher than to the 2 eV bulk [64]. This might be due to the structure defects, that have changed the phase, strain and size of nanoparticles during heat treatment that led to the increase of band gap [74]. When Fe_2_O_3_ added on chitosan, the band gap became lower as compared to the individual band gap. It can be due to the increased of crystallite size attributed to the confinement effects that related to the rise amount of orbitals participating in the formation of valence bands and covalent bands through orbital overlap [75]. Thus, this showed that defects and confinement effects have a huge impact on the optical properties of a composite.

### 3.4. Optical-Based Sensing of Hg^2+^

**Figure 10:**
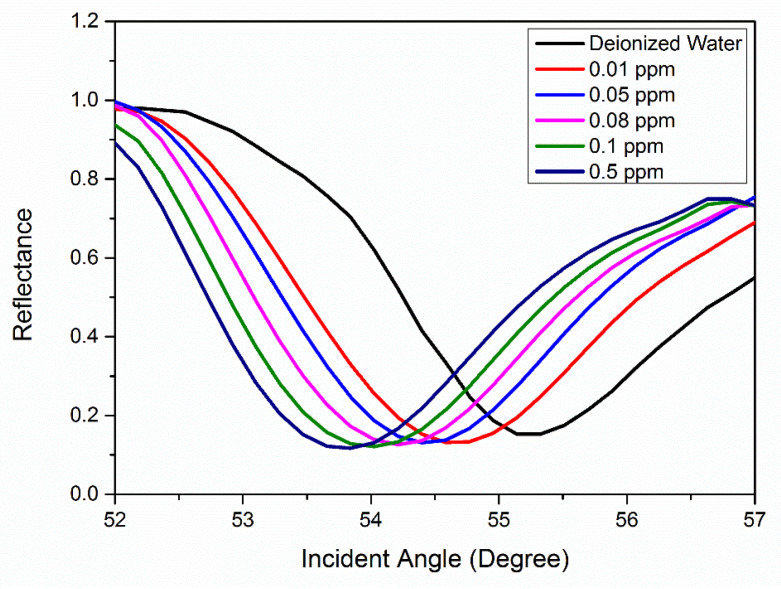
SPR curves for Cs/Fe_2_O_3_ thin film in contact with deionized water and Hg^2+^ solution with a concentration of 0.01–0.5 ppm.

The optical sensing based on surface plasmon resonance (SPR) phenomenon was conducted by using Cs/Fe_2_O_3_ thin film to identify the SPR angle for deionized water as a control experiment. The SPR angle of 55.225° was further applied to compare the SPR angle for different concentration of Hg^2+^ solution ranged from 0.01 to 0.5 ppm. The SPR reflectively curves for Cs/Fe_2_O_3_ thin film in contact with the different concentration of Hg^2+^ are shown in Figure 10. It can be seen that the SPR curves of Hg^2+^ solution shifted from 0 to 0.5 ppm as compared with the deionized water SPR curve. The SPR angle for 0.01, 0.05, 0.08, 0.1 and 0.5 ppm of Hg^2+^ were 54.615°, 54.398°, 54.212°, 54.027° and 53.836°, respectively, with the corresponding error of ±0.001° (the resolution of the stepping motor of the SPR). Overall, it was observed that the SPR shifted to the left with increasing concentration of Hg^2+^ solution. This finding can be attributed to the increase in binding between analyte–ligand, which resulted in the change of refractive index as well as the thickness of the Cs/Fe_2_O_3_ sensing layer [76,77,78,79]. Hence it is confirmed that Cs/Fe_2_O_3_ thin film has an affinity with Hg^2+^ and can be integrated with SPR optical-based sensing method for detection of Hg^2+^.

## 4. Conclusions

In this study, a Cs/Fe_2_O_3_ thin film was successfully developed using the spin coating technique. The functional groups analysis from the FTIR results confirmed the correlation between chitosan and γ-Fe_2_O_3_, with the peak intensity of Cs/Fe_2_O_3_ clearly increasing after the sorption of chitosan and Fe_2_O_3_ at C–H stretching, C–C bond and O=C=O stretching. Next, the AFM result showed that the thin film was homogenous when the surface of chitosan on the thin film was covered by Fe_2_O_3_. Besides, the UV-Vis results confirmed that the Cs/Fe_2_O_3_ thin film had the lowest absorbance value compared its individual thin films with an optical band gap of 4.013 eV. The incorporation Cs/Fe_2_O_3_ thin film with the optical-based sensing method using the surface plasmon resonance technique provided positive response to the Hg^2+^ solution of different concentrations. This result demonstrated the enormous ability of Cs/Fe_2_O_3_ thin film for optical sensing of Hg^2+^ as low as 0.01 ppm. 

## Figures and Tables

**Table 1:** Chitosan based material by surface plasmon resonance (SPR) for the detection of Hg^2+^.

Ref.	Sensing Layer	LOD
[38]	MMW chitosan (glutaraldehyde-crosslinked)	2.49 µM
[52]	Polypyrrole-chitosan conducting polymer composite	2.50 µM
[53]	Chitosan/graphene oxide	0.50 µM
[54]	Polypyrrole-chitosan/nickel-ferrite nanoparticles	1.94 µM

Ref.: reference. LOD: limit of detection.
